# Pericardial effusion caused by accidently placing a Micra transcatheter pacing system into the coronary sinus

**DOI:** 10.1186/s12872-021-02266-1

**Published:** 2021-09-23

**Authors:** Xueying Chen, Jingfeng Wang, Yixiu Liang, Yangang Su, Junbo Ge

**Affiliations:** Department of Cardiology, Shanghai Institute of Cardiovascular Disease, National Clinical Research Center for Interventional Medicine, Zhongshan Hospital, Fudan University, 180 Fenglin Road, Shanghai, 200032 China

**Keywords:** Case report, Leadless pacemaker, Pericardial effusion, Coronary sinus

## Abstract

**Background:**

Leadless pacemaker has been acknowledged as a promising pacing strategy to prevent pocket and lead-related complications. Although rare, cardiac perforation remains a major safety concern for implantation of Micra transcatheter pacing system (TPS).

**Case presentation:**

A 83-year-old female with low body mass index (18.9 kg m^−2^) on dual anti-platelet therapy, was indicated for Micra TPS implantation due to sinus arrest and paroxysmal atrial flutter. The patient developed mild pericardial effusion during the procedure since the delivery catheter was accidentally placed into the coronary sinus for several times. Cardiac perforation with moderate pericardial effusion and pericardial tamponade was detected 2 h post-procedure. The patient was treated with immediately pericardiocentesis and recovered without further invasive therapy.

**Conclusion:**

Pericardial effusion caused by accidently placing a delivery catheter into the coronary sinus is rare but should be carefully considered in Micra TPS implantation, especially for those with periprocedural anti-platelet therapy.

**Supplementary Information:**

The online version contains supplementary material available at 10.1186/s12872-021-02266-1.

## Introduction

Recently, leadless pacemaker has emerged as a new pacing strategy to avoid pocket and lead-related complications as compared with conventional pacemaker [1]. However, though the incidence of major complications was demonstrated to be low to 1.51% in Micra transcatheter pacing system (TPS) (Medtronic, Minneapolis, MN, USA) [2], cardiac perforation remains a major safety concern with the incidence of about 0.13–1.3% in studies [2, 3]. Herein, we presented a case of cardiac perforation with pericardial tamponade caused by accidently placing the delivery catheter into the coronary sinus.

## Case presentation

**Fig. 1:**
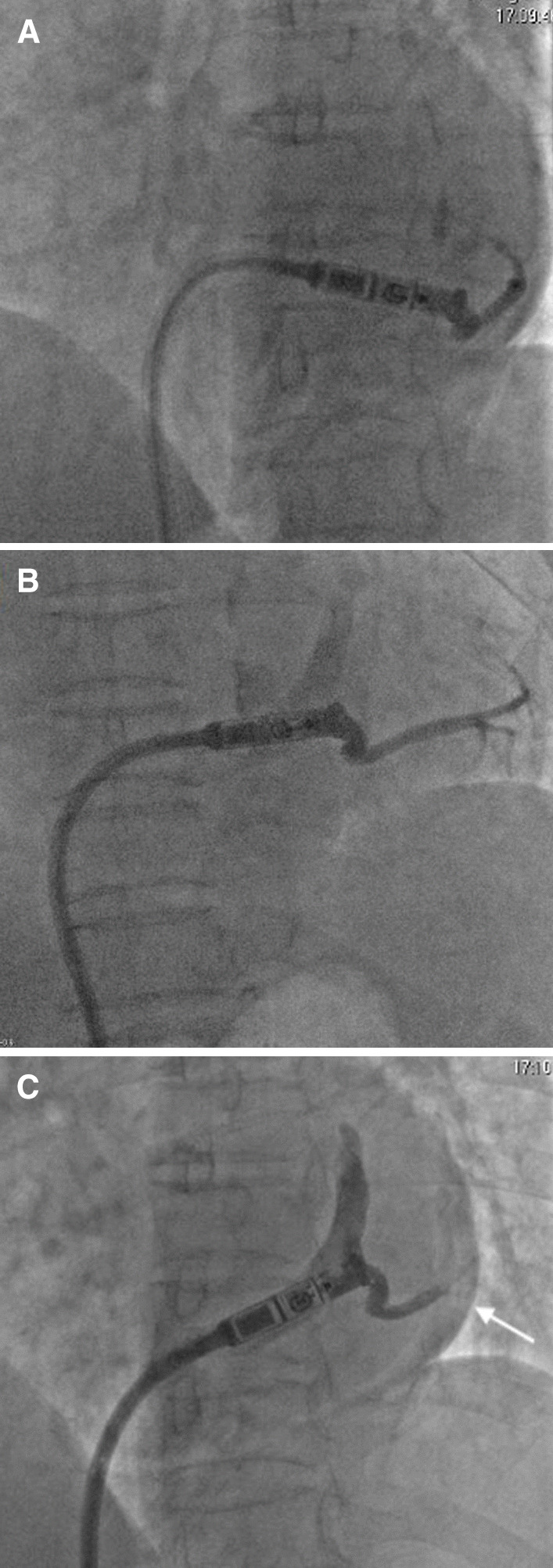
Angiography showing Micra TPS placing into the coronary sinus: **A** At poster-anterior view; Pericardial effusion (arrow) at left anterior oblique view for the first attempt (**B**) and the second attempt (**C**)

**Fig. 2:**
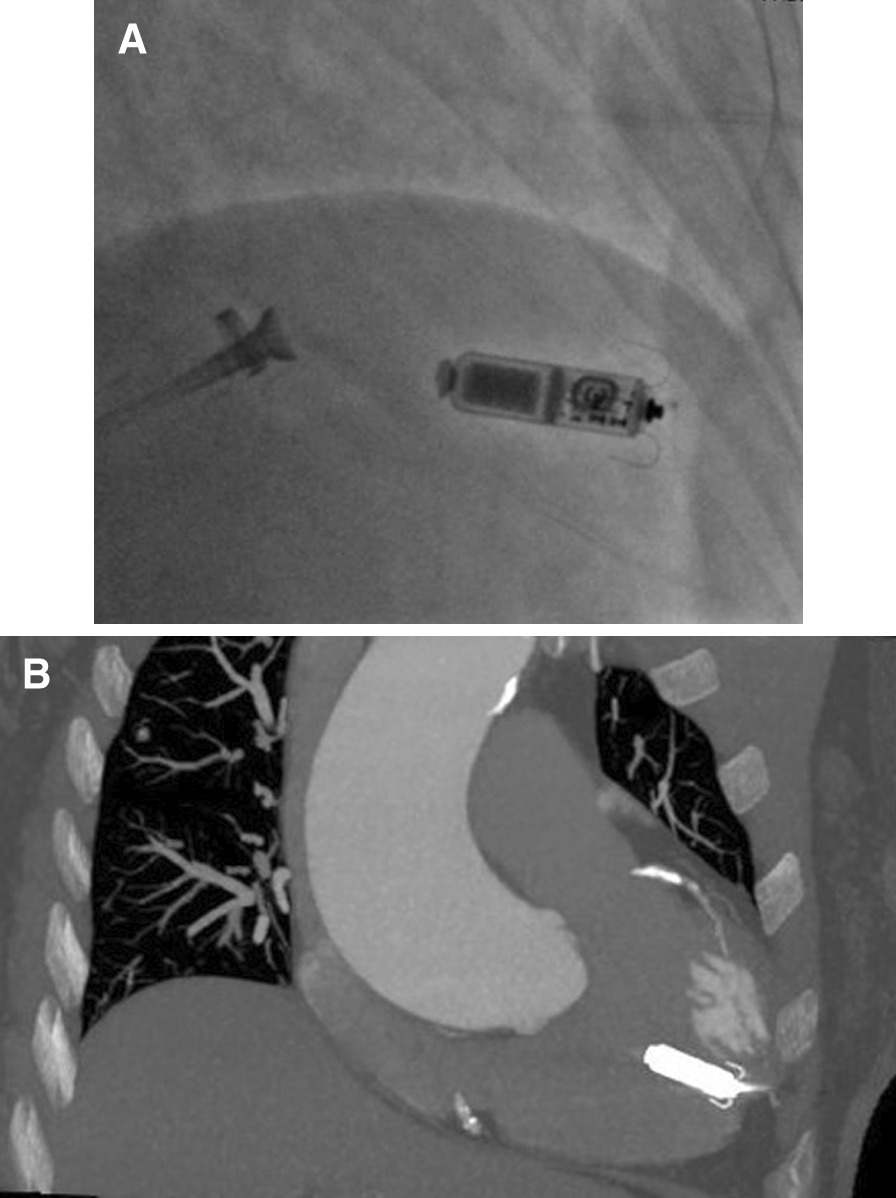
The images showing the location of Micra leadless pacemaker: **A**. After deployment at right anterior oblique view; **B**. CT scan after implantation indicating the leadless pacemaker locating at the apex of right ventricular

**Fig. 3:**
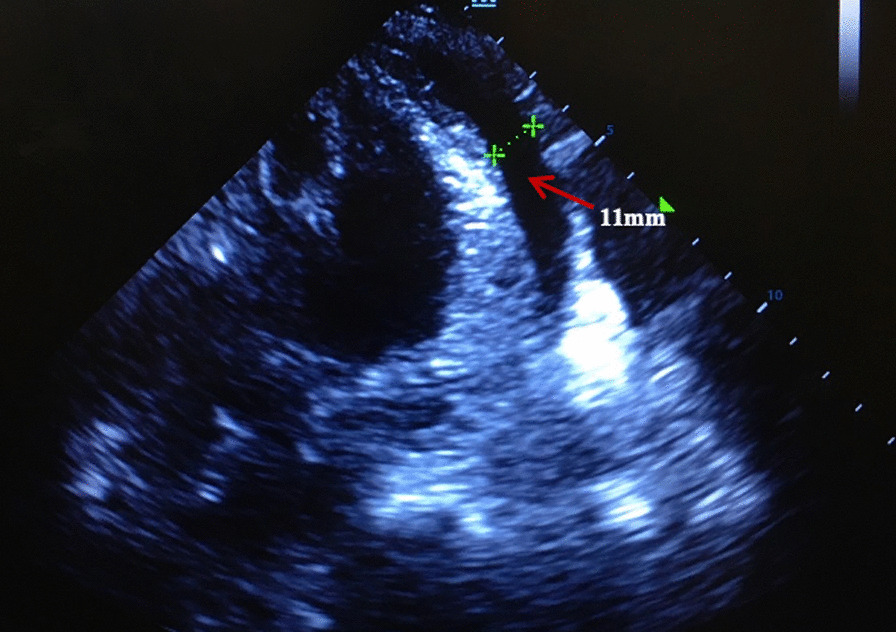
The echocardiogram showing pericardial effusion (red arrow) during diastolic phase mainly distribution around the posterior wall of left ventricle

A 83-year-old female (height, 148 cm; weight, 41.5 kg; body mass index, 18.9 kg∙m^−2^) suffered from sinus arrest of 5 s with paroxysmal atrial flutter and was admitted to our hospital for leadless pacemaker implantation. The patient received percutaneous coronary intervention with 2 stents implantation 1 month before admission and dual anti-platelet therapy of aspirin (100 mg/day) and clopidogrel (75 mg/day) were continued to the procedure day. During the procedure, the patient was received intravenous heparin 50u/kg before Micra TPS was introduced into the right femoral vein. Then the delivery catheter was directed across the tricuspid valve but it was accidently performed into the coronary sinus for several times. The device cup was advanced into the posterior branch of coronary sinus as confirmed by angiography (Fig. 1)(Additional files 1, 2). Simultaneously, pericardial effusion was detected at the left anterior oblique view (Fig. 1B, C). The delivery catheter was immediately pulled back to the right atrium. The patient was asymptomatic and remained hemodynamically stable (blood pressure 134/76 mmHg). After adjusting the direction of the catheter, it was finally successfully performed into the right ventricle and Micra leadless pacemaker was deployed at the apex of right ventricle (Fig. 2) with stable pacing parameters (R wave amplitude, 9 mV; threshold, 0.38 V/0.24 ms; impedance, 1000Ω). The patient’s condition remained stable until 2 h post-procedure, she was found pericardial tamponade with blood pressure dropped to 75/58 mmHg and heart rate increased to 96 beats per minute. Medium amount of pericardial effusion mainly distribution around the posterior wall of left ventricle was confirmed by echocardiogram (Fig. 3). The patient was emergently received pericardiocentesis and drainage of 270 ml bloody fluid. The symptoms were immediately relieved with blood pressure rise to 130/80 mmHg. Her dual anti-platelet therapy was suspended until no evidence of distinct pericardial effusion was detected after the drainage tube removal. Although no definite evidence was announced for application of rivaroxaban 5 mg in preventing embolic events, aspirin (100 mg/day) and rivaroxaban (5 mg/day) were initially prescribed afterwards concerning balance between ischemia (prevention of thrombosis in stents and thromboembolism due to atrial flutter) and bleeding risk in this elder female with low body mass index and extremely fragile state. The patient was discharged without pericardial effusion reconfirmed by echocardiogram. At 1-month follow-up, the pacing parameters remained stable and the patient was prescribed clopidogrel (75 mg/day), aspirin (100 mg/day) and rivaroxaban (5 mg/day) without pericardial effusion by echocardiogram. And the patient was followed-up without evidence of pericardial effusion, bleeding, thrombosis or thromboembolism at 3-month after discharge (Table 1).

**Table 1:** A time line from admission to 3 months after discharge

Time line	Patient’s condition	Pacing parameters	Treatment	Medications
On admission	Normal	–	–	Aspirin (100 mg/day) and clopidogrel (75 mg/day)
During the procedure	Asymptomatic pericardial effusion with hemodynamically stable	Stable	–	–
At 2 h post-procedure	Pericardial tamponade	Stable	Pericardiocentesis and drainage of 270 ml bloody fluid	Stopped anti-platelet therapy for 5 days
On 6th day post-procedure	No pericardial effusion	Stable	Drainage tube removal	Aspirin (100 mg/day) and rivaroxaban (5 mg/day)
At 1-month follow-up	No pericardial effusion	Stable	–	Aspirin (100 mg/day), clopidogrel (75 mg/day) and rivaroxaban (5 mg/day)
At 3-month follow-up	No pericardial effusion, bleeding, thrombosis or thromboembolism	Stable	–	Aspirin (100 mg/day), clopidogrel (75 mg/day) and rivaroxaban (5 mg/day)

## Discussion

Due to the different fixation way, cardiac perforation remains one of the severe complications of leadless pacemaker. It is recommended to implant leadless pacemaker at the septum of the right ventricle to minimize the incidence of cardiac perforation [2, 4], though it is not easy to be achieved in all patients, especially in small hearts or cor pendulum (drop hearts) cases. According to the literatures [2, 3], the risk factors for cardiac perforation in leadless pacemaker included female, low body mass index, history of myocardial infarction and lung diseases. Therefore, each patient should be carefully estimated before implantation, especially in cases with these risk factors.

### Possible reasons for cardiac perforation of the present case

It is challenging to implant the leadless pacemaker in small-size heart cases since the shape of the delivery catheter is fixed. In this case, it is not easy to perform Micra TPS across the tricuspid valve and accidently place it into the branch of coronary sinus after multiple attempts. Other than cardiac injury by the fixation apparatus after deployment of Micra, cardiac perforation resulting from the delivery catheter against the ventricular wall has also been illustrated. Togashi [5] et al. reported a case of subclinical cardiac perforation caused by the edge of the device cup penetrating into the ventricular wall prior to the deployment of the leadless pacemaker. Another 91-year-old female reported by Hai [4] et al. developed cardiac perforation due to contrast injection against the RV anterior wall before verification of sheath location. The cause of pericardial effusion in the present case was probably the coronary vein injury by the edge of the device cup, since pericardial effusion was detected by angiography when the catheter was advanced into the coronary sinus before releasing Micra (Fig. 1). The pericardial effusion aggravated and pericardial tamponade occurred post-procedure probably due to dual anti-platelet therapy before procedure together with anti-coagulation of heparin during procedure.

Learning curve of the operator might be another possible reason for the complication. As a tertiary center, 6 electrophysiologists are specialized in pacemaker implantation in 2 electrophysiology rooms, with > 70 Micra procedures per year and > 1600 other kinds of pacemakers implantation per year, respectively. Though the operator of this case is well-trained and has independently implanted more than 50 cases of Micra before, Micra implantation is relatively a new procedure in our center since 2019 as compared with conventional pacemaker procedures.

### How to avoid cardiac perforation induced by coronary vein injury

To avoid such complication, carefully advancing the Micra TPS at both posterior and left anterior oblique view are helpful to distinguish Micra TPS locating at coronary sinus or right ventricle. If the Micra TPS was performed into the coronary sinus accidently as confirmed at left anterior oblique view, mildly pulled back the delivery catheter without angiography might decrease the risk of coronary vein injury. Once pericardial effusion occurs, protamine, a rapidly acting antidote for heparin, should be used at the end of the procedure to avoid pericardial effusion aggravation. On the other hand, in terms of short half-life period, bivalirudin might be more suitable than heparin for peri-implantation anti-coagulation in patients on dual anti-platelet therapy to reduce the bleeding risk.

## Conclusion

Pericardial effusion caused by accidently placing a delivery catheter into the coronary sinus is rare but should be carefully considered in Micra TPS implantation, especially for those with periprocedural anti-platelet therapy.

## Supplementary Information

**Additional file 1: Video 1:** Intra-procedural angiography showed that the delivery catheter of Micra was directed into the coronary sinus.

**Additional file 2: Video 2:** A fluoroscopy video clip showed the level of advancement of Micra inside the posterior branch of the coronary sinus

## Data Availability

The data used in the literature review are available from the corresponding author on reasonable request.

## Abbreviations

TPS: Transcatheter pacing system
RV: Right ventricular
